# What Internet Services Would Patients Like From Hospitals During an Epidemic? Lessons From the SARS Outbreak in Toronto

**DOI:** 10.2196/jmir.7.4.e46

**Published:** 2005-08-03

**Authors:** Carlos A Rizo, Doina Lupea, Homayoun Baybourdy, Matthew Anderson, Tom Closson, Alejandro R Jadad

**Affiliations:** ^3^University of TorontoDepartment of Health PolicyManagement and EvaluationTorontoONCanada; ^2^University Health NetworkTorontoONCanada; ^1^Centre for Global eHealth InnovationUniversity Health Network and University of TorontoTorontoONCanada

**Keywords:** Severe acute respiratory syndrome, communicable diseases, emerging, information services, Internet, public health, questionnaires

## Abstract

**Background:**

International health organizations and officials are bracing for a pandemic. Although the 2003 severe acute respiratory syndrome (SARS) outbreak in Toronto did not reach such a level, it created a unique opportunity to identify the optimal use of the Internet to promote communication with the public and to preserve health services during an epidemic.

**Objective:**

The aim of the study was to explore patients’ attitudes regarding the health services that might be provided through the Internet to supplement those traditionally available in the event of a future mass emergency situation.

**Methods:**

We conducted “mask-to-mask” surveys of patients at three major teaching hospitals in Toronto during the second outbreak of SARS. Patients were surveyed at the hospital entrances and selected clinics. Descriptive statistics and logistic regression models were used for the analysis.

**Results:**

In total, 1019 of 1130 patients responded to the survey (90% overall response rate). With respect to Internet use, 70% (711/1019) used the Internet by themselves and 57% (578/1019) with the help of a friend or family member. Of the Internet users, 68% (485/711) had already searched the World Wide Web for health information, and 75% (533/711) were interested in communicating with health professionals using the Internet as part of their ongoing care. Internet users expressed interest in using the Web for the following reasons: to learn about their health condition through patient education materials (84%), to obtain information about the status of their clinic appointments (83%), to send feedback to the hospital about how to improve its services (77%), to access screening tools to help determine if they were potentially affected by the infectious agent responsible for the outbreak (77%), to renew prescriptions (75%), to consult with their health professional about nonurgent matters (75%), and to access laboratory test results (75%). Regression results showed that younger age, higher education, and English as a first language were predictors of patients’ interest in using Internet services in the event of an epidemic.

**Conclusion:**

Most patients are willing and able to use the Internet as a means to maintain communication with the hospital during an outbreak of an infectious disease such as SARS. Hospitals should explore new ways to interact with the public, to provide relevant health information, and to ensure continuity of care when they are forced to restrict their services.

## Introduction

International public health organizations and officials around the world are bracing for a pandemic [1-6]. Reports highlight that conditions for the global dissemination of avian flu [7-8] or influenza [9-10] are already emerging. It has been suggested that government officials may be underestimating the threat and that more aggressive allocation of resources is needed to minimize the potential devastation that a new pandemic could cause [5].

The 2003 severe acute respiratory syndrome (SARS) outbreak caused major disruption of hospital services in affected countries [11-15] and provided unique insights in terms of how to react to larger epidemics or full-blown pandemics (see also Textbox 1). Emergency containment and preventive measures required hospitals to cancel most clinics and operative procedures [16-18]. At the University Health Network (UHN) in Toronto, rigorous surveillance measures were instituted, and hospital access was restricted to single entrance points for staff and patients. Visits to the hospital were limited or prohibited. Working closely with public health authorities to maximize the impact of these measures, affected hospitals established Command Centres to prevent the spread of infection and provide information to staff and the public [19]. Whenever appropriate, hospital staff with no direct clinical responsibilities were encouraged to work remotely. Websites and Internet-based messaging systems were implemented to notify staff about policies and procedures instituted to contain the outbreak [20]. Telephone-based call centres made thousands of calls per day to alert patients about changes in clinic schedules and to provide nonurgent medical advice and prescription refills.

Although effective, telephone-based communication proved to be a resource-intensive solution that may not be sustainable in the event of a more widespread epidemic or external disaster. Considering that most Canadians have access to the Internet [21], it could be argued that the Internet may have the potential to facilitate information flow between hospitals and many of their patients during a crisis, complementing or replacing other means of communication. To our knowledge, no studies have explored patients’ views with regard to the use of the Internet as a complementary or alternative form of communication during an epidemic. This study was designed to explore these views and the specific Internet-based services patients would like to have available in the event of a future outbreak.

**University Health Network–during and after SARS**

- **Communication with staff and outpatients:** Shortly after the beginning of the SARS outbreak, the hospital enhanced its communication capability to ensure the flow of real-time information for public and staff. This process included mass voice mails from the Chief Executive Officer informing staff of the current status and expanding videoconferencing and Web mail services. Web mail traffic increased by 300% in the first days of the outbreak. Meanwhile, hits to the UHN website almost doubled from January to July 2003, from 273269 to 548108, respectively. Interestingly, website hits continued to increase throughout the year and never returned to pre-SARS figures. The corporate intranet was also made accessible over the Internet, enabling UHN to communicate with staff and ensure work continuity despite the environmental restrictions.
- **Communication support for inpatients:** UHN contracted the services of TLContact CarePages, enabling patients to send updates to family and friends over the Internet [22].
- **Remote access to UHN’s electronic health record:** The electronic health record was made accessible to physicians over the Internet. Since then, physicians can consult patients’ charts remotely. Through an application known as Patient Results Online (PRO), the hospital is providing its clinicians with real-time access to patient results. PRO also allows access to lab results stored in partner hospitals.
- **Electronic scheduling:** UHN developed an electronic scheduling application to reduce unnecessary patient travel, improve patient satisfaction, and reduce waiting lists. Implemented across all three UHN sites, this system allows electronic access to scheduling and contact information. In the event of a hospital closure, staff working offsite will be able to access schedules and contact patients, removing the need for intense telephone booking, rescheduling, and cancellations.
- **Infection control screening and surveillance:** UHN developed a SARS screening tool that is currently built into its registration screens. In the event of an outbreak, the surveillance software can be activated and the hospital can track where patients have been in the hospital and where they are headed.
- **Insight-alerting system software:** This software monitors patient information and alerts health care professionals about critical situations in real time. The software checks information as it is entered into the system and is supported by “rules” to detect potentially dangerous clinical situations. This will be beneficial during an outbreak as the system can detect a positive diagnosis of an infectious disease and alert health care providers by pager, email/Blackberry, the Web, or fax.

## Methods

After obtaining permission from the UHN’s Command Centre and approval from the institutional Research Ethics Board, we conducted a cross-sectional survey at the single access points of each of the hospitals comprising UHN (Toronto General Hospital, Toronto Western Hospital, and Princess Margaret Hospital) and six of the ambulatory clinics that remained open during July 2003.

### Patient Survey

**Figure 1:**
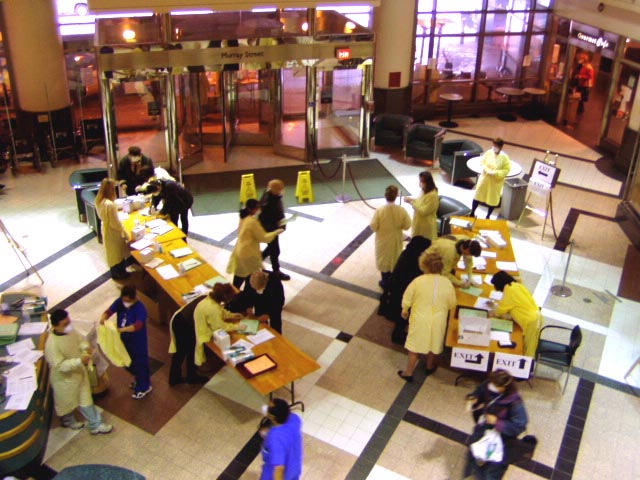
Screening during SARS at the University Health Network

The survey was based on the core questions of the UHN e1000 study, a cross-sectional survey exploring the patterns of Internet use among patients and providers associated with the UHN. The e1000 survey [23] is a longitudinal study that has gathered cross-sectional data twice (January 2001 and April 2002) in seven ambulatory clinics. For this study, we continued assessing patients’ patterns of Internet use for general and health-related purposes, adding questions regarding their opinions about services they would like to receive through the Internet in the event of hospital closure, clinic postponement, or procedure cancellation due to an outbreak. Respondents could also suggest other uses for the Internet in a health-related context. Additionally, the survey examined the influence of patients’ demographics (ie, gender, age, level of education completed, first language, and country of birth) on awareness and use of the Internet in general and on seeking health-related information in particular. The Command Centre recommended that the survey should not take more than 3 minutes—much shorter than initially planned—in order to avoid congestion in the screening lineups by the entrance doors (Figure 1).

To alleviate possible anxiety during the crisis, only 9 questions with categorical responses were asked, in addition to those capturing demographic information (Multimedia Appendix 1).

### Study Logistics

**Figure 2:**
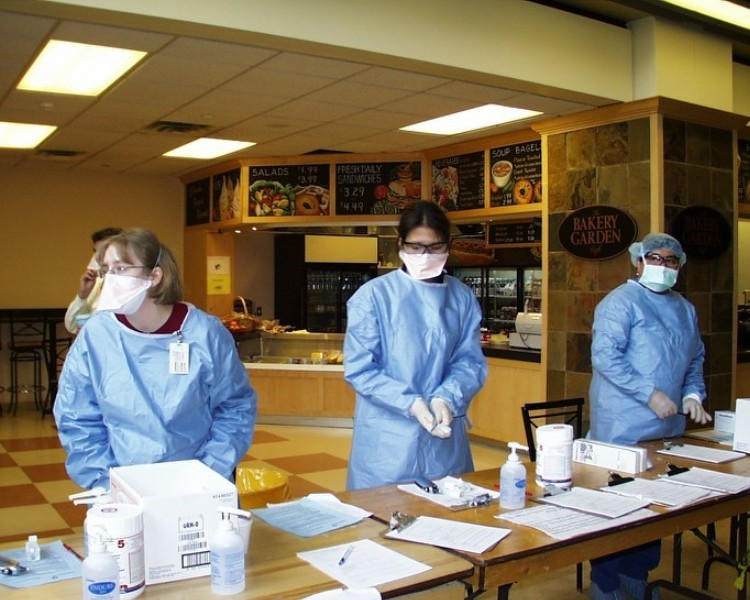
Protective gear for staff and interviewers during the SARS outbreak at UHN

The initial plan to survey all patients entering the hospital after being screened for SARS (approximately 1000 patients daily per hospital) proved to be unrealistic due to the cumbersome process of registration and screening that created additional challenges for those people approaching patients. For three days, we surveyed patients after they passed the entrance screening points, and we concluded that the clinics’ waiting rooms would be a more appropriate venue for recruiting. Only adult ambulatory patients were approached. Ten trained multilingual interviewers conducted the surveys in English after obtaining verbal consent. The interviewers and patients were required to wear full protective gear, which meant “mask-to-mask” rather than face-to-face communication (Figure 2).

With no face recognition and to avoid re-approaching the same patients, interviewers used colored stickers on the masks of patients to identify those already invited to participate in the survey.

### Data Protection

Contact information provided voluntarily by patients was stored in a secure database on the Centre for Global eHealth Innovation servers. Hard copies of the surveys were stored in locked cabinets and were accessible to researchers for analysis only at the Centre.

### Statistical Analysis

Descriptive statistics were gathered for each of the answers. Multiple logistic regression analysis was used to assess the effect of the sociodemographic variables on patients’ views of Internet usage for specific services in the event of a mass emergency. We used stepwise, forward, and backward methods with all variables to specify which ones stayed in the model. A cutoff *P* value of .2 was chosen for variable elimination. Then, we applied the enter method on those variables to force all variables into the equation. Results reported in the tables are based on the remaining variables only and include odd ratios (OR) and 95% confidence intervals. A *P* value less than .05 was considered statistically significant. The variables that did not stay in the model at first step are marked with "NS." All statistical analyses were conducted using the SAS System for Windows, release 8.02 (SAS Institute Inc, Cary, NC).

### Quality

To ensure better reporting, we used the relevant items of the Checklist for Reporting Results of Internet E-Surveys (CHERRIES) [24]. Although CHERRIES is designed for online surveys, we applied the relevant domains of the checklist to our survey.

## Results

A total of 1130 patients were approached, and 1019 chose to complete the survey, giving an overall participation rate of 90%. Refusal rate was initially higher at the entrances of the hospitals during the first three days of the survey (response rate 78%, 309/396 respondents approached) than later at the clinics that remained open (response rate 97%, 710/734 respondents approached). The most frequent reasons for declining were being late for a clinic appointment (50%), frustration and exhaustion due the long lineup to enter the hospital (20%), lack of interest (20%), and inability to speak English (10%).

### Internet Use for Health Information and Interest in Communicating with Health Professionals

We found that 91% of patients were aware of the Internet (926/1019) and that 70% used the Internet (711/1019) by themselves and 57% (578/1019) with the help of a friend or family member. Of the Internet users, 68% (485/711) had already searched the World Wide Web for health information, and 75% (533/711) were interested in using the Internet to communicate with health professionals as part of their ongoing care.

Table 1 shows the demographic characteristics of all respondents, those with Internet access, and by survey location (entry point or clinic). Overall, there was a balanced gender representation of Internet users in our sample, with 44% (313/711) women and 45% (320/711) men. The majority of Internet users were in the 21 to 40 (32%, 224/711) and 41 to 60 (40%, 281/711) age categories. Almost half (42%, 300/711) of the Internet users had college or undergraduate education, 3 out of every 4 Internet users (558/711) spoke English as their first language, and 64% of them (453/711) were born in Canada. These demographic proportions are comparable for all users and for users at the entry doors or clinics.

There were no statistically significant differences between Internet users surveyed at entry doors and clinics with respect to age (*P* = .14), English as a first language (*P* = .90), or country of birth (*P* = .54). However, there were significant differences with respect to education (*P* < .001) and gender (*P* = .005). Regardless of the survey location, when all users were combined there was no significant gender difference.

**Table 1:** Demographic characteristics*

**Variables**	**All Respondents** (n = 1019)	**Internet Users** (n = 711)	**Internet Users at Entry Doors** (n = 197)	**Internet Users at Clinics** (n = 514)
**Gender**				
Male Female	44% (451/1019) 42% (433/1019)	45% (320/711) 44% (313/711)	38% (74/197) 54% (106/197)	48% (246/514) 40% (207/514)
**Age**				
< 21 21-40 41-60 > 60	2% (21/1019) 24% (246/1019) 37% (378/1019) 24% (249/1019)	3% (21/711) 32% (224/711) 40% (281/711) 15% (108/711)	1% (3/197) 27% (53/197) 44% (87/197) 18% (35/197)	3% (18/514) 33% (171/514) 38% (194/514) 14% (73/514)
**Education Completed**				
Elementary High school College/Undergraduate Postgraduate	5% (49/1019) 34% (346/1019) 35% (354/1019) 12% (124/1019)	1% (9/711) 29% (203/711) 42% (300/711) 17% (119/711)	1% (3/197) 19% (37/197) 48% (95/197) 23% (46/197)	1% (6/514) 32% (166/514) 40% (205/514) 14% (73/514)
**First Language**				
English Other	72% (734/1019) 28% (285/1019)	78% (558/711) 22% (153/711)	78% (154/197) 22% (43/197)	79% (404/514) 21% (110/514)
**Country of Origin**				
Canada Other	57% (583/1019) 43% (436/1019)	64% (453/711) 36% (258/711)	62% (122/197) 38% (75/197)	64% (331/514) 36% (183/514)

^*^ Percentages may not add to 100% for each variable due to missing responses.

### Internet Services During Mass Emergencies

In the event of a future outbreak, Internet users expressed interest in accessing the Internet to learn about their health condition through patient education materials (84%, 594/711), to obtain information about the status of their clinic appointment (83%, 590/711), to send feedback to the hospital about how to improve its services (77%, 549/711), to access screening tools to help determine if they were affected by the infectious agent responsible for the outbreak (77%, 544/711), to renew prescriptions (75%, 535/711), to consult with their health professional about nonurgent matters (75%, 536/711), and to obtain laboratory results (75%, 534/711). Respondents had the opportunity to suggest other uses for the Internet, and 10% (70/711) chose to do so. Their most frequent suggestion was the ability to communicate with family members, as visits were restricted. Others wanted to use the Internet to access their electronic health record, participate in virtual support groups, replace certain follow-up visits with online consultations, and find information on drug compatibility or clinical trials.

Statistically significant demographic predictors for interest in specific Internet services among Internet users are shown in Table 2.

**Table 2:** Logistic regression of demographic factors (independent variables: rows) predicting interest in specific Internet services (dependent variable: columns) among Internet users (n = 711)

**Odds Ratio (95% CI) (*P* value)**													
	**Communicate with health care professionals using Internet(Q5)**	**Find out the status of clinic appointment(Q6a)**	**Request a prescription refill(Q6b)**	**Obtain lab results(Q6c)**		**Consult about non urgent matters(Q6d)**		**Learn through patient education program(Q6e)**		**Send feedback about improving services(Q6f)**		**Access screening tools(Q6g)**	
**Age**													
< 40	1.98 (1.16–3.37) (*P* = .01)	2.37 (1.30–4.29) (*P* = .03)	2.12 (1.24–3.62) (*P* = .02)	1.98 (1.16–3.36) (*P* = .07)		1.96 (1.18–3.25) (*P* = .18)		2.27 (1.26–4.09) (*P* = .07)		2.51 (1.44–4.38) (*P* = .002)		2.05 (1.21–3.47) (*P* = .12)	
41-60	1.38 (0.83–2.29) (*P* = .92)	1.93 (1.09–3.43) (*P* = .32)	1.76 (1.05–2.95) (*P* = .35)	1.84 (1.09–3.09) (*P* = .18)		2.23 (1.34–3.70) (*P* = .02)		2.18 (1.23–3.86) (*P* = .12)		1.61 (0.95–2.72) (*P* = .94)		2.21 (1.32–3.72) (*P* = .04)	
> 60 (RC)	1	1	1	1		1		1		1		1	
**Education**													
High school or less (RC)	1	1	1	1		1		1		1		1	
College/ University	1.79 (1.20–2.65) (*P* = .003)	2.34 (1.48–3.69) (*P* < .001)	2.19 (1.48–3.24) (*P* < .001)	2.04 (1.38–3.02) (*P* <.001)		1.67 (1.13–2.48) (*P* = .01)		1.55 (0.98–2.46) (*P* = .06)		1.72 (1.13–2.61) (*P* = .01)		NS	
**English First Language**													
No (RC)	1	1	1	1		1		1		1		1	
Yes	1.87 (1.13–3.08) (*P* = .01)	2.45 (1.41–4.28) (*P* = .001)	2.13 (1.13–4.02) (*P* = .02)	2.73 (1.46–5.09) (*P* = .001)		1.36 (0.81–2.28) (*P* = .24)		1.38 (0.76–2.51) (*P* = .28)		1.96 (1.16–3.31) (*P* = .01)		NS	
**Gender**													
Female (RC)	1	1	1	1		1		1		1		1	
Male	0.75 (0.51–1.11) (*P* = .16)	NS	NS	NS		NS		NS		1.33 (0.87–2.01) (*P* = .18)		NS	
**Born in Canada**													
No (RC)	1	1	1	1		1		1		1		1	
Yes	NS	NS	0.58 (0.34–0.99) (*P* = .048)	0.54 (0.31–0.94) (*P* = .03)		NS		NS		NS		NS	

RC = reference category

NS = nonsignificant factors (*P* values > .2)

Internet users (Table 2) younger than 40 years were significantly more likely to be interested in communicating with health professionals over the Internet, finding the status of their appointments, requesting prescription refills, and sending feedback to the hospital about improving services than those 41 to 60 years old. Interestingly, the odds for those aged 41 to 60 interested in consulting about nonurgent matters were significantly higher than for younger patients. All Internet users with college or university education were significantly more likely than participants with high school or elementary education to be interested in services provided through the Internet in case of a mass emergency, except for accessing screening tools or learning through patient education materials. Respondents with English as their first language were more likely to be interested in receiving services though the Internet in the event of a mass emergency. The likelihood of being interested in Internet services was not significant for gender.

The detailed results for the populations at the entry doors and at the clinics are provided in Multimedia Appendix 2. At the entry doors (Table 3 in Multimedia Appendix 2), the younger population was more likely than the older population to be interested in communicating with health professionals using the Internet or in sending feedback to the hospital. Patients between 41 and 60 years old were more likely to be interested in finding the status of a clinic appointment through the Internet than patients over 60 years. Although this trend was also detected among patients younger than 40, the result was not statistically significant. The odds of being interested in electronic communication and consulting about nonurgent matters were higher for college- and university-educated individuals than for those with lower levels of education. Men were slightly more interested than women in accessing test results over the Internet, and people born in Canada were more likely to be interested in requesting a prescription refill or obtaining a lab result than people born outside of Canada.

At the clinics (Table 4 in Multimedia Appendix 2), participants younger than 40 years were more likely than their older counterparts to be willing to send feedback to the hospital. Participants with undergraduate education were significantly more interested in finding the status of their clinic appointment, requesting a prescription refill, obtaining a laboratory result, and sending feedback to the hospital than people with high school or less. English speakers were more likely to be interested in all Internet services except for accessing patient education materials.

Overall, younger age, higher education, and English as a first language were predictors of interest in using Internet services in the event of a pandemic, with a few exceptions.

## Discussion

### Principal Results

Four people in Toronto died of SARS, while hundreds were infected around the world. However, the SARS outbreak pales in comparison to a full-blown pandemic. For instance, the bubonic plague killed more than 130 million people, while the Spanish flu pandemic of 1918 killed more than 30 million. In Philadelphia, the 1793 yellow fever outbreak took the lives of more than 4000 people. Today, research suggests that the world is due for a pandemic [1-6] of unprecedented proportions that could dramatically disrupt the activities of health organizations.

The 2003 SARS outbreak challenged the way in which health organizations deal with public health crises. Although the classic outbreak control measures (infection control, contact tracing, quarantine, etc) were used in order to overcome new obstacles, such as high volume of air travel, increased media attention, and generalized panic, alternative methods of communication and collaboration to overcome them were required.

Similar to what happened during the anthrax scare [25], the Internet provided a powerful way to offer information about the outbreak to patients and members of the public [26,27]. It also enabled data sharing and collaboration among health professionals and organizations around the world [28,29]. The Internet, however, may have not been used to its full potential as a means of communication between hospitals and the public during the SARS outbreak. Hospital communication with the public mostly relied on unidirectional mass media releases on the radio, television, newspapers, and Internet, except for an isolated case in which hotlines were used in a temperature-monitoring campaign [30]. Hospital staff relied on the telephone to communicate with health care providers who were quarantined in their homes. The hospital did not participate in activities to support quarantined members of the public, as this was done by public health officials.

Our results suggest that most patients are willing and able to use the Internet as a means to preserve and complement hospital information and communication services during an outbreak of an infectious disease such as SARS.

The results of this study are consistent with previously conducted surveys at UHN during non-SARS times. These results are related only to the proportion of patients using the Internet for general and health purposes. Earlier iterations indicate that 60% of respondents have used the Internet for general purposes and 69% for retrieving health information [23]. During SARS, the proportion of UHN patients using the Internet was higher (69%). This increase may be due to a combination of factors (timing of the survey, higher awareness and adoption, chance) and not necessarily due to the epidemic. Previous results showed that three of every four patients wanted to use email and websites as means of communication with health providers. This result is comparable with the one obtained during the SARS outbreak (75%). Consistent with previous results, patients more likely to be aware of and use the Internet were younger, more educated, and spoke English as their first language [23]. Unlike our previous surveys, the current one shows that older patients (> 60 years) were more likely to be interested in communicating about nonurgent matters with health professionals than their younger counterparts (41 to 60 years). This may be due to a combination of increased familiarity with the Internet over the previous two years in a population that faces chronic conditions and the realization that some face-to-face meetings may be replaced with online alternatives [26].

We conducted a systematic review of the literature looking for surveys of patients in relation to the type of services desired but did not find similar enough studies to justify a comparison with our SARS survey (data not shown).

There are many other potential uses for the Internet as a means of communication if hospitals and clinics were disabled by a new outbreak. Members of the public with Internet access who are quarantined may use it to get answers to nonurgent questions related to the infectious disease or to receive reassurance that they are managing their health properly [31]. Patients whose appointments are changed could receive customized information about their own care (eg, normal test results) or obtain prescription refills via simple text email messages. Family members of hospitalized patients, unable to visit their loved ones, may receive information about their loved ones’ health status through patient-specific websites or blogs [22]. Teleconference booths could also be set up in the community so that hospitalized patients or individuals in isolation could continue to be in touch with their loved ones if the latter do not have easy access to the Internet.

Harnessing the power of the Internet in the event of a new outbreak, and particularly during a pandemic, will require changes at the hospital level that need to be gradually introduced during “new normal” times. At the very least, as part of the patients’ registration process, hospitals should collect data regarding patients’ choice for communication method (telephone, email, or both) in the event of an outbreak.

The SARS crisis underscored many opportunities for the use of Internet-mediated communication to extend the continuum of care outside of hospital walls, even under normal circumstances. Embracing the Internet as an integral part of clinical care, however, will require changes in legislation, funding structures, and flexible work patterns to enable health professionals to use it [32].

The findings of our survey highlight the need for timely, relevant, valid, feasible, and substantiated options to maintain communication lines with the public during crises that disable hospitals. We are aware that Internet access is not yet universal, but it certainly could be very valuable for the large subset of the population that uses it [33], while enabling more efficient allocation of resources to support those who require other communication modalities.

With the current increased risk of pandemics and bioterrorist attacks, it is essential to put in place the mechanisms necessary to use the Internet effectively and efficiently in order to reduce the impact of these crises on the health system and the public at large.

### Limitations

The special circumstances under which these surveys were conducted presented several design, execution, and data analysis limitations.

#### Design Considerations

The sample chosen was one of convenience. Due to constraints inherent to the emergency, it was difficult to ensure that all individuals attending the hospital at any given date had the same probability of being selected. The difficulty of obtaining an up-to-date list of patients visiting the hospital limited our ability to establish an accurate denominator. Therefore, the likelihood of the sample being representative of the population attending the hospital during the second outbreak of SARS is unknown. To reduce the evident sampling bias, a number of random sampling techniques could have been used. Assuming that the population at selected clinics was captive and had patients with similar health conditions, cluster sampling may have been indicated.

#### Execution Considerations

At the hospital entry points, some interviewers noted that respondents were rushing and may have fallen into a rut (a “response set”), continuing to give the same response unthinkingly. This was particularly evident for question 6, which may have elicited repeated identical responses (ie, yes). In addition, the number of refusals at the single point entrances was higher than previously obtained at UHN [23]. To mitigate these considerations, the surveyors moved to the clinics that remained open, and they were encouraged to pause between asking questions.

#### Analysis Considerations

Comparisons of participants from the three participating hospitals and an assessment of users versus non-Internet users were outside the scope of this study. Furthermore, the patient’s health condition was not collected. For future studies, these elements will be considered.

## Multimedia Appendix 1

Study survey.

## Multimedia Appendix 2

Additional tables (Tables 3 and 4) showing the results of the populations at the entry doors and clinics.
