# Supplementary material for: What Internet Services Would Patients Like From Hospitals During an Epidemic? Lessons From the SARS Outbreak in Toronto
Source: J Med Internet Res. 2005 Aug 3;7(4):e46. doi: 10.2196/jmir.7.4.e46 (PMC1550678; doi:10.2196/jmir.7.4.e46)
Supplement: Supplementary file 1 [file jmir_v7i4e46_app1.pdf]

Centre for Global eHealth Innovation
UHN e1000 Project - Patient Survey 2003

Patient Family Friend | Interviewer: ____________________

Date: __DD / MM / YYYY_____ **Clinic you will visit today:**______________ | TGH TWH PMH

Q1. Have you ever heard of the Internet or World Wide Web?

Yes *(Continue with Q2)* No *(Skip to Patient Demographics)*

Q2. Have you ever used the Internet?

Yes No

Q3. Has a family or friend used the Internet on your behalf?

Yes No *(Skip to Q5)* Unsure

Q4. From where have you, or someone on your behalf, accessed the Internet? *(Check as many as apply)*

At work At home At a friend or family member's house

At hospital or clinic At the public library Elsewhere: ______________________

Q5. Would you be interested to communicate with healthcare professionals using the Internet (e.g., e-mail) as part of your ongoing care, either by yourself or with the help of someone close to you?

Yes No Unsure

Q6. In the event that the hospital closes clinics or postpones procedures/surgeries during situations like SARS, and if these were available, would you, either by yourself or with the help of someone close to you, use the Internet to access the following: *(Check as many as apply)*

a. Find out the status of your clinic appointment	Yes	No	Unsure
b. Request a prescription refill	Yes	No	Unsure
c. Obtain lab results	Yes	No	Unsure
d. Consult with a hospital health professional about non-urgent matters	Yes	No	Unsure
e. Learn about a disease through patient education information	Yes	No	Unsure
f. Send feedback to the hospital about how to improve its services	Yes	No	Unsure
g. Access screening tools	Yes	No	Unsure
h. Other *(please specify)*: ______________________________	Yes	No	Unsure

Q7. Do you, or does someone close to you, use the Internet to find information about your health?

Yes No Unsure

Q8. Have you ever shared any of the information found on the Internet with a healthcare provider?

Yes No Unsure

Q9. Would you be willing to be involved in future projects to improve the use of the Internet in healthcare?

Yes No Unsure

If you would like to be involved in future projects, please provide your contact information.
Rest assured that the information provided will be kept confidential and used only by UHN research staff.

Name: | **E-mail:**

Address: | **Telephone : ( ) -**

Gender: M F | **Age:** <21 21-40 41-60 >60

Education: Elementary School High School College/Undergraduate Post-Graduate

Is English your first language? Yes No **which one?**____

Were you born in Canada? Yes No **where?** ____

SARS and Internet Survey
