# Supplementary material for: What Internet Services Would Patients Like From Hospitals During an Epidemic? Lessons From the SARS Outbreak in Toronto
Source: J Med Internet Res. 2005 Aug 3;7(4):e46. doi: 10.2196/jmir.7.4.e46 (PMC1550678; doi:10.2196/jmir.7.4.e46)
Supplement: Supplementary file 2 [file jmir_v7i4e46_app2.pdf]

Multimedia Appendix to Rizo et al.
What Internet Services Would Patients Like From Hospitals During an Epidemic? Lessons From the SARS Outbreak in Toronto.
J Med Internet Res 2005;7(4) <URL: http://www.jmir.org/2005/4/>

Table 3. Logistic regression–potential services of interest to Internet users surveyed *at entry doors*

	**Odds Ratio (95% CI) (*P* value)**							
**Variable**	**Interest in communication with health care professionals using Internet (Q5)**	**Find out the status of clinic appointment (Q6a)**	**Request a prescripti on refill (Q6b)**	**Obtain lab results (Q6c)**	**Consulting about non-urgent matters (Q6d)**	**Learn through patient educatio n program (Q6e)**	**Send feedback about improving services (Q6f)**	**Access screening tools (Q6g)**
**Age**								
40	3.42 (1.20–9.76) (*P* = .03)	5.96 (1.47–24.04) (*P* = .31)	3.63 (1.24–3.62) (*P* = .15)	NS	2.59 (0.95–7.04) (*P* = .75)	3.75 (1.61–12.12) (*P* = .16)	3.85 (1.18–12.50) (*P* = .04)	2.01 (0.76–5.33) (*P* = .46)
41-60	1.77 (0.72–4.32) (*P* = .90)	8.96 (2.27–35.40) (*P* = .05)	3.45 (1.35–8.81) (*P* = .15)	NS	5.11 (1.85–14.07) (*P* = .01)	3.20 (1.17–8.78) (*P* = .27)	1.77 (0.70–4.44) (*P* = .80)	2.23 (0.89–5.55) (*P* = .23)
> 60 (RC)	1	1	1	1	1	1	1	1
**Education**								
High school or less (RC)	1	1	1	1	1	1	1	1
College/ University	2.84 (1.29–6.25) (*P* = .009)	NS	NS	NS	2.69 (1.12–6.46) (*P* = .02)	NS	NS	NS
**English First Language**								
No (RC)	1	1	1	1	1	1	1	1
Yes	NS	NS	NS	NS	NS	NS	NS	NS
**Gender**								
Female (RC)	1	1	1	1	1	1	1	1
Male	0.59 (0.27–1.26) (*P* = .17)	NS	NS	0.34 (0.14–0.78) (*P* = .01)	NS	NS	NS	NS
**Born in Canada**								
No (RC)	1	1	1	1	1	1	1	1
Yes	NS	NS	NS	NS	NS	NS	NS	NS

RC = reference category; NS = nonsignificant *P* values that did not show up in the final model

Table 4. Logistic regression–potential services of interest to Internet users surveyed *at clinics*

Variable	Interest in communication with healthcare professionals using Internet (Q5)	Find out the status of clinic appointment (Q6a)	Request a prescription refill (Q6b)	Obtain lab results (Q6c)	Consulting about non-urgent matters (Q6d)	Learn through patient education program (Q6e)	Send feedback about improving services (Q6f)	Access screening tools (Q6g)
	**Odds Ratio (95% CI) (*P* value)**							
**Age**								
40	NS	1.97 (1.00–3.86) (*P* = .06)	1.93 (1.02–3.64) (*P* = .054)	2.30 (1.21–4.35) (*P* = .053)	1.77 (0.96–3.23) (*P* = .20)	1.99 (0.99–3.97) (*P* = .23)	2.38 (1.26–4.49) (*P* = .01)	2.21 (1.17–4.18) (*P* = .14)
41-60	NS	1.46 (0.76–2.80) (*P* = .87)	1.47 (0.79–2.74) (*P* = .79)	2.05 (1.09–3.88) (*P* = .21)	1.74 (0.95–3.18) (*P* = .24)	1.94 (0.97–3.88) (*P* = .19)	1.69 (0.91–3.14) (*P* = .68)	2.33 (1.23–4.42) (*P* = .07)
> 60 (RC)	1	1	1	1	1	1	1	1
**Education**								
High school or less (RC)	1	1	1	1	1	1	1	1
College/ University	1.47 (0.94–2.29) (*P* = .09)	1.86 (1.14–3.03) (*P* = .01)	2.43 (1.56–3.79) (*P* < .001)	2.40 (1.51–3.80) (*P* < .001)	1.41 (0.90–2.20) (*P* = .12)	1.61 (0.96–2.71) (*P* = .06)	1.60 (1.01–2.55) (*P* = .045)	1.32 (0.81–2.14) (*P* = .25)
**English First Language**								
No (RC)	1	1	1	1	1	1	1	1
Yes	1.77 (0.89–3.51) (*P* = .09)	2.46 (1.32–4.57) (*P* = .004)	2.72 (1.26–5.85) (*P* = .01)	4.08 (1.86–8.92) (*P* < .001)	1.90 (1.05–3.43) (*P* = .03)	1.89 (0.96–3.72) (*P* = .06)	2.65 (1.46–4.78) (*P*= .001)	2.14 (1.15–3.98) (*P* = .01)
**Gender**								
Female (RC)	1	1	1	1	1	1	1	1
Male	NS	NS	NS	1.40 (0.88–2.22) (*P* = .14)	NS	NS	NS	NS
**Born in Canada**								
No (RC)	1	1	1	1	1	1	1	1
Yes	1.55 (0.89–2.70) (*P* = .11)	NS	0.58 (0.31–1.11) (*P* = .10)	0.55 (0.28–1.08) (*P* = .08)	NS	NS	NS	NS

RC = reference category; NS = nonsignificant *P* values that did not show up in the final model
